# Anti-Obesity Sodium Tungstate Treatment Triggers Axonal and Glial Plasticity in Hypothalamic Feeding Centers

**DOI:** 10.1371/journal.pone.0039087

**Published:** 2012-07-03

**Authors:** Marta Amigó-Correig, Sílvia Barceló-Batllori, Guadalupe Soria, Alice Krezymon, Alexandre Benani, Luc Pénicaud, Raúl Tudela, Anna Maria Planas, Eduardo Fernández, Maria del Carmen Carmona, Ramon Gomis

**Affiliations:** 1 Diabetes and Obesity Laboratory, Institut d’investigacions Biomèdiques August Pi i Sunyer, Endocrinology and Nutrition Unit-Hospital Clínic, Barcelona, Spain; 2 Centro de Investigación Biomédica en Red de Diabetes y Enfermedades Metabólicas Asociadas, Barcelona, Spain; 3 University of Barcelona, Barcelona, Spain; 4 Department of Brain Ischemia and Neurodegeneration, Institut d’Investigacions Biomèdiques de Barcelona, Consejo Superior de Investigaciones Científicas, Institut d’investigacions Biomèdiques August Pi i Sunyer, Barcelona, Spain; 5 Experimental 7T MRI Unit, Institut d’investigacions Biomèdiques August Pi i Sunyer, Barcelona, Spain; 6 Taste and Food Science Center, UMR 6265-CNRS, 1324-INRA, University of Bourgogne, Dijon, France; 7 Centro de Investigación Biomédica en Red de Bioingeniería, Biomateriales y Nanomedicina, Group of Biomedical Imaging of the University of Barcelona, Barcelona, Spain; 8 Bioengineering Institute and Centro de Investigación Biomédica en Red de Bioingeniería, Biomateriales y Nanomedicina, Miguel Hernández University, Elche, Spain; Montreal Diabetes Research Center, Canada

## Abstract

**Objective:**

This study aims at exploring the effects of sodium tungstate treatment on hypothalamic plasticity, which is known to have an important role in the control of energy metabolism.

**Methods:**

Adult lean and high-fat diet-induced obese mice were orally treated with sodium tungstate. Arcuate and paraventricular nuclei and lateral hypothalamus were separated and subjected to proteomic analysis by DIGE and mass spectrometry. Immunohistochemistry and *in vivo* magnetic resonance imaging were also performed.

**Results:**

Sodium tungstate treatment reduced body weight gain, food intake, and blood glucose and triglyceride levels. These effects were associated with transcriptional and functional changes in the hypothalamus. Proteomic analysis revealed that sodium tungstate modified the expression levels of proteins involved in cell morphology, axonal growth, and tissue remodeling, such as actin, CRMP2 and neurofilaments, and of proteins related to energy metabolism. Moreover, immunohistochemistry studies confirmed results for some targets and further revealed tungstate-dependent regulation of SNAP25 and HPC-1 proteins, suggesting an effect on synaptogenesis as well. Functional test for cell activity based on c-fos-positive cell counting also suggested that sodium tungstate modified hypothalamic basal activity. Finally, *in vivo* magnetic resonance imaging showed that tungstate treatment can affect neuronal organization in the hypothalamus.

**Conclusions:**

Altogether, these results suggest that sodium tungstate regulates proteins involved in axonal and glial plasticity. The fact that sodium tungstate could modulate hypothalamic plasticity and networks in adulthood makes it a possible and interesting therapeutic strategy not only for obesity management, but also for other neurodegenerative illnesses like Alzheimer’s disease.

## Introduction

Western countries are currently facing a dramatic increase in the incidence of metabolic diseases, including obesity and type-2 diabetes. In basic terms, obesity develops when energy intake exceeds energy utilization, breaking the energy balance [1]. Deciphering biological mechanisms that control food intake and energy homeostasis could help find a way to stop the epidemic. In healthy animals, food intake is permanently adjusted to fit cumulative energy expenditure. This energy homeostasis is finely tuned by endocrine and neural mechanisms. In this regard, specific brain areas sense modifications in peripheral metabolic parameters, such as blood glucose, free fatty acids, and circulating insulin and leptin levels, and elicit appropriate behavioral and autonomic responses.

The hypothalamus is highly involved in this physiologic feedback. This diencephalic structure contains distinct nuclei, among which arcuate (ARC), lateral (LHA) and paraventricular (PVN) nuclei have key roles in the control of energy homeostasis [2]. The ARC contains at least two neuronal subpopulations controlling energy balance, which are components of a highly structured hypothalamic network [3] and project to other hypothalamic areas, such as PVN and LHA [4], [5]. This organization is essential for the long-term maintenance of energy homeostasis, as well as aberrant hypothalamic development, which especially affects neuronal projections from ARC to PVN, and is associated with a predisposition to diet-induced obesity [6]. In addition, impaired connectivity in specific neuronal networks located within the ARC could be one reason for hyperphagia in leptin-deficient mice [7].

Interestingly, the hypothalamus remains “plastic” in adulthood, meaning that neuronal networks in this structure can undergo functional or morphological remodeling. Recently, it has been shown that some metabolic signals could act as trophic factors to induce not only the organization of the hypothalamus during development [8], but also its continuous re-organization in adult mice [7], [9]. On the contrary, excessive caloric intake may be a negative signal for neuronal plasticity in adulthood, while caloric restriction potentiates remodeling of brain areas [10]. These observations enhance the idea of neuronal plasticity as a central player to maintain brain circuitry for the adequate regulation of energy balance [11].

Sodium tungstate is a neutral salt of tungsten. In aqueous solution it acts as a phosphatase inhibitor, changing the catalytic activity of these enzymes [12], [13]. Several animal studies have highlighted the possible role of sodium tungstate in regulating different aspects of energy homeostasis in rodents [14], [15]. We have demonstrated that sodium tungstate also plays an important role in the hypothalamus, activating leptin’s pathway [16] and regulating the gene expression of the main neuropeptides involved in the control of food intake: neuropeptide Y (NPY), agouti related peptide (AgRP) and proopiomelanocortin (POMC) [14]. Furthermore, it has been described that sodium tungstate can inactivate glycogen synthase-3β (GSK3β) (a kinase that hyperphosphorylates tau in Alzheimer’s disease) in neuroblastoma cells and in the rat cerebral cortex [17], showing its involvement in other neurological areas.

Here, we investigated whether a chronic treatment with sodium tungstate can rewire hypothalamic networks, which may be involved in restoring eating behavior and improving the peripheral metabolism of obese animals. For this purpose, we first analyzed the molecular signature of hypothalamic plasticity after tungstate administration through proteomic and immunohistochemistry analyses of different hypothalamic nuclei from mice. We then scanned the brains of mice after tungstate administration using magnetic resonance imaging to explore a putative tungstate-related structural plasticity.

## Materials and Methods

### Animals, Surgery and Drug Administration

All animal experiments were carried out in accordance with the European Communities Council directive (86/609/EEC) and the practical guide for the care and use of laboratory animals published by the Spanish National Research Council, which specifically approved this study. For this study, 8-week-old male C57BL6/J mice and 8-week-old male C57BL6/J diet-induced obesity (DIO) mice (fed with a high-fat diet from weaning) (Harlan laboratories, Indianapolis, IN, USA) were used. The animals were kept in a temperature-controlled room (22±1°C) on a 12-h light-dark cycle with free access to food and water. In accordance with the experiments, mice were fed with either a standard diet (22.4% fat, 60.9% carbohydrate and 16.7% protein; 2.9 Kcal/g; R04T25, Safe, France) or a high-fat diet (60% fat, 20% carbohydrate and 20% protein; 5.24 Kcal/g; D12492, Research Diets, New Brunswick, NJ, USA). Between 10–12 weeks of age, half of the lean and DIO mice groups were treated with 2 g/l sodium tungstate (Na_2_WO_4_X2H_2_O; Sigma-Aldrich, St. Louis, MO, USA) in drinking water for 14 days. The calculated average dose was 130 mg/kg/day (water intake per day 2.6 ml regardless of the group). During the entire experimental period, water intake, food intake, and body weight of all animals were periodically recorded. For tissue collection, mice were killed by decapitation. Brains were quickly removed and hypothalamus dissected. 12-week-old male Wistar rats (Charles River Laboratories, Sta. Perpètua de la Mogoda, Spain) were used for intracerebroventricular injections (icv) as previously described [16].

### Hypothalamus Dissection and Nucleus Enrichment

Brains were immersed for 10 min in 2 ml of ice-cold preservative medium (200 mM sucrose, 28 mM NaHCO_3_, 2.5 mM KCl, 7 mM MgCl_2_, 1.25 mM NaH_2_PO_4_, 0.5 mM CaCl_2_, 1 mM L-ascorbate, and 8 mM D-glucose, pH 7.4), complemented with a 10% anti-protease cocktail (Complete Mini, Roche Diagnostics, Indianapolis, IN, USA). Hypothalamus dissection and nucleus enrichment were performed as previously described [18]. In brief, 0.5 mm slices using a vibrating microtome were prepared and placed in ice-cold preservative medium. From bregma −0.46 to −2.46, different nuclei were identified and cut, and pieces were then put into separate microcentrifuge tubes and stored at −80°C. Mean tissue weights were 3 mg for ARC and PVN and 6 mg for LHA, respectively.

### Hypothalamic Nucleus Proteomics and DIGE Analyses

DIGE analyses of hypothalamic nuclei were performed as previously described [19]. Samples were taken from the following groups: untreated lean animals (UL) (n = 4); lean animals treated with sodium tungstate (TL) (n = 4); untreated obese animals (UO) (n = 4); and obese animals treated with sodium tungstate (TO) (n = 4). These samples were homogenized in a buffer containing 7 M urea, 2 M thiourea, 2% (w/v) CHAPS, 65 mM DTT, 0.5% (v/v) IPG buffer, and 10 mM sodium orthovanadate. Proteins were extracted for 30 min at 4°C, and lysates were clarified by centrifugation at 14000 rpm at 4°C for 30 min. Protein extracts were precipitated using a 2D clean-up kit (GE Healthcare, Pollards Wood, United Kingdom) and resuspended in a buffer containing 30 mM Tris, 7 M urea, 2 M thiourea, 4% CHAPS, pH 8.5. Finally, protein content was quantified using an RC DC protein assay kit (Bio-Rad, Hercules, CA, USA). Samples were minimally labeled with Cy3 or Cy5 fluorescent dyes (50 µg of protein/400 pmol of dye) during 30 min at 4°C and multiplexed, as described in table S1. An internal standard (pool from all the samples present) labeled with Cy2 was included in each gel. 2D electrophoresis was performed using IPG strips pH 3–10 as the first dimension and 10% acrilamide as the second dimension. Fluorescence images of the gels were acquired on a Typhoon 9400 scanner (GE Healthcare). Cy2, Cy3, and Cy5 images for each gel were scanned at 488/520-, 532/580-, and 633/670-nm excitation/emission wavelengths, respectively, at 100-µm resolution. Image analysis was performed using DeCyder version 6.5 software (GE Healthcare).

### Protein Digestion, Mass Spectrometry, Protein Identification and Analysis

The same gels used for DIGE analyses were used as preparative gels and were silver-stained using an MS-compatible protocol [20]. Differentially expressed proteins were selected, excised from the different gels silver stained, and in-gel digested with trypsin at 37°C overnight (Promega, Madison, WI, USA). Peptide extraction was performed, and ZipTip concentrating and desalting was done as described previously [21], [22]. Peptides were analyzed in a MALDI-TOF/TOF 4700 Proteomics Analyzer (Applied Biosystems, Foster City, CA, USA) in the reflector/delayed extraction mode, and ESI-Q-TOF Global (MicromasS, Cary, NC, USA). Peptide masses from MS analyses and their fragments obtained from MS/MS spectra were combined and submitted to Sequence Query Mascot software (Matrix Science, Boston, MA, USA). Searches were performed using Swiss-Prot as the database and mammals as taxonomy. Error tolerated was 0.07-Da for peptide mass, 0.8-Da for fragment mass (MS/MS), and a single trypsin missed cleavage. Proteins were considered identified when they had a significant (p<0.05) Mascot score. Ingenuity Pathways Knowledge Base was used to analyze the predominant interaction networks and high-level functions of differentially expressed proteins (www.ingenuity.com).

### 1D and 2D Western Blots

For 2D gels, samples were homogenized in 7 M urea, 2 M thiourea, 2% (w/v) CHAPS, 65 mM DTT, 0.5% (v/v) IPG buffer, and 10 mM sodium orthovanadate and were quantified. 50 µg were then loaded on IPG strips (7 cm, pH 3–10) and run on a Protean IEF cell at 7300 V/h. The second dimension was run as described previously [23] in 10% acrylamide gels. For 1D gels from brain proteins, protein isolation was performed in a protein lysis buffer containing: Hepes 10 nM; KCl 10 nM; Sucrose 240 mM; EDTA 2 mM; DTT 0.5 mM; phosphatase inhibitors (sodium orthovanadate 10mM, PMSF 0.5mM, and sodium pyrophosphate 149 mM); and 1× protease inhibitor cocktail (Sigma-Aldrich). Tissues were homogenized and centrifuged, and supernatants were collected. Proteins were quantified and subjected to separation in an 8% acrylamide gel. All gels were transferred to polyvinylidene fluoride (PVDF) membranes at 100 V for 1 h 30 minutes, and probed with 1/1000 dilution of rabbit anti-pCRMP-Thr^514^, anti-CRMP2, anti-Phospho-p44/42 MAPK, anti-pSTAT3-Y^705^ (Cell signalling, Beverly, MA, USA), and anti-beta actin (Sigma-Aldrich).

### Immunohistochemistry, Analysis, and Quantification of Staining Intensity

Animals were anesthetized and transcardially perfused with 15 ml of cold PBS with 0.1% heparin followed by 50 ml of cold 4% (w/v) paraformaldehyde in PBS. Brains were carefully removed from the skull and soaked overnight in the fixative solution (4% buffered paraformaldehyde), 24 h on glucose 30%-containing 1×PBS, dehydrated-deffated, and embedded in paraffin. Tissue blocks were cut into 5-µm coronal sections using a rotating microtome and were serially collected. Representative sections, 1 among 10, containing ARC were selected for immunohistochemical analysis. To reduce nonspecific reactions, sections were incubated for 20 min in a blocking solution of 10% normal donkey serum (Jackson Immunoresearch Laboratories, West Grove, PA, USA) in PBS with 0.5% Triton X-100. The sections were incubated overnight with primary antibodies: c-fos, syntaxin 1A (HPC-1) 1∶100 (Santa Cruz Biotechnology, Santa Cruz, CA, USA); β-tubulin 1∶200, synaptosomal-associated protein 25 (SNAP-25) 1∶100, collapsing response mediator protein 2 (CRMP2) 1∶50 (Abcam, Cambridge, United Kingdom); glial fibrillary acidic protein (GFAP) 1∶100 (Sigma-Aldrich); and neurogenic differentiation factor 1 (NeuroD1) 1∶200 (Chemicon, Temecula, CA, USA). Each section was incubated for 2 hours in the secondary antibody, biotinylated anti-goat made in donkey IgG 1∶100 (Jackson Immunoresearch Laboratories). Finally, the sections were incubated in 1 mg/ml streptavidin Alexa Fluor 555 conjugates (Molecular Probes, Invitrogen, Carlsbad, CA, USA) in PBS with 0.5% Triton X-100. Preparations were then placed under cover slips using a water-based mounting agent for fluorescent preparations (Vectashield, Vector, Burlingame, CA, USA). Images were analyzed using the computer-assisted image analysis program ImageJ (developed and maintained by the National Institutes of Health, Bethesda, MD, USA) and a set of customized macros. The overall immunofluorescence labeling was standardized for an accurate quantification of the staining following the methods of Taylor and Levenson [24]. To account for variations in staining intensity due to immunohistochemical methods, intensity was normalized in the range of 0 to 255. Counting of positive cells was performed at a magnification of 40× by the same experimenter who was blinded to the animal treatment. Two different sections of the ARC per animal, anatomically matched across all animals studied and distributed between Bregma −1.06 and −2.06, were counted and summed to give one number per animal.

### Magnetic Resonance Imaging (MRI)

MRI experiments were conducted on a 7.0 T BioSpec 70/30 horizontal animal scanner (Bruker BioSpin, Ettlingen, Germany), equipped with a 12 cm inner diameter actively shielded gradient system (400 mT/m). The receiver coil was a phased array surface coil for mouse brain. Animals were placed in a supine position in a plexiglas holder with a nose cone for administering anaesthetic gases (isofluorane in a mixture of 30% O_2_ and 70% CO_2_) and were fixed using a tooth bar, ear bars and adhesive tape. Tripilot scans were used for accurately positioning the animal’s head in the isocenter of the magnet. Diffusion Tensor Imaging (DTI) acquisition was performed by using an echo planar imaging (EPI)-based DTI sequence, applying TR = 6000 ms, TE = 32.11 ms, 4 segments, 3 averages, b-value = 1000, 30 different diffusion directions, 5 A0 images, slice thickness = 1 mm, number of slices = 14, FOV = 1.5×1.5×0.9 mm^3^, and matrix size = 128×128×18 pixels, which resulted in a spatial resolution of 0.12×0.12 mm in 0.5 mm slice thickness.

DTI maps of tensor diffusivity, fractional anisotropy (FA), main diffusivity (MD), axial diffusivity (λ_1_) and radial diffusivity (λ_2_+ λ_3_/2) were calculated using Paravision 5.0 software (Bruker Biospin), and custom programs written in MATLAB (The MathWorks, Inc., Natick, MA, USA), as previously described [25], [26]. Regions of interest (ROI) were individually drawn for each subject by an experienced neurobiologist blinded to the treatments, overlaying the MRI images with the digital Paxinos and Watson rat brain atlas. The PVN region was drawn at −0.82 and −1.32 mm from Bregma, and ARC was drawn at −1.46 and −1.96 mm from Bregma. The mean values of ROIs for each diffusion index and nucleus calculated were considered for statistical analysis.

### Statistical Analysis

Data are presented as mean±SEM. A one-way analysis of variance followed by Tukey’s multiple-comparison test was used to assess the statistical difference between groups. When appropriate, an unpaired t-test was performed.

## Results

### Sodium Tungstate Effects on Metabolic Parameters

**Figure 1:**
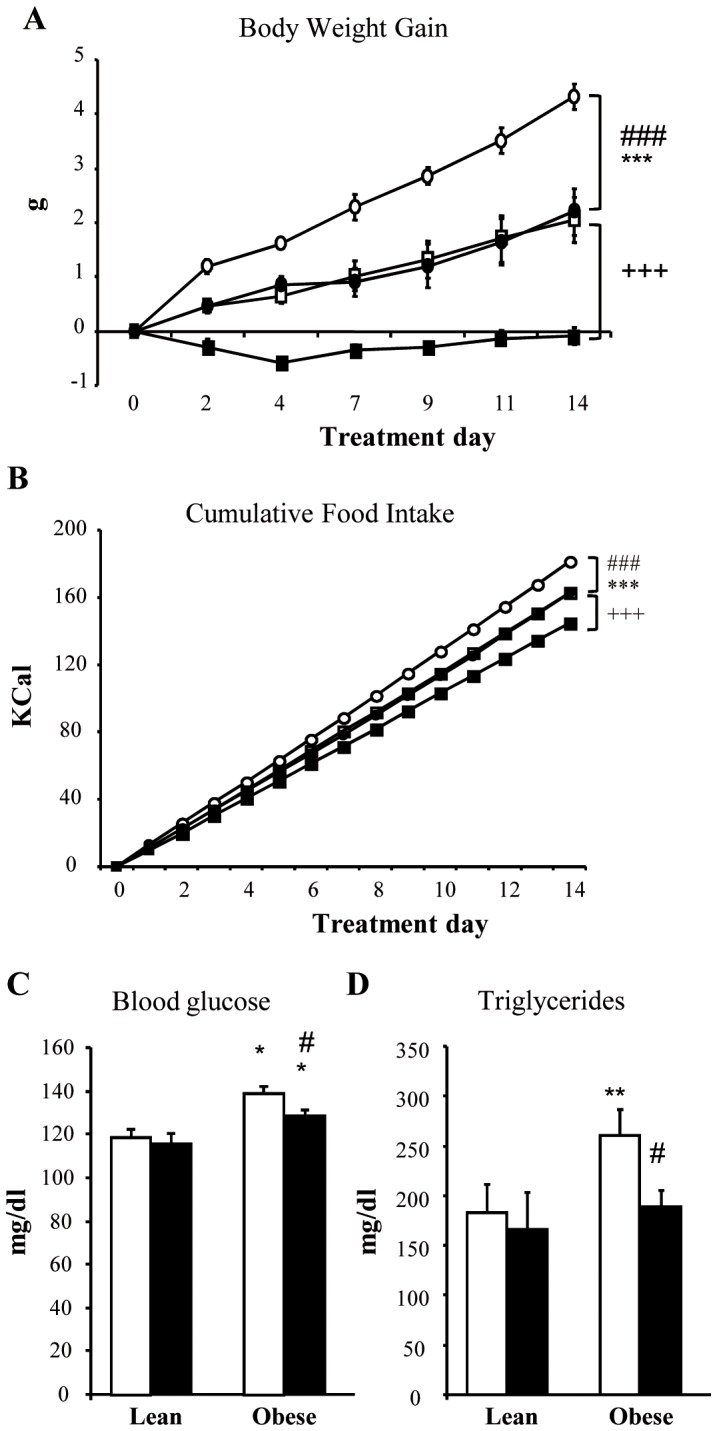
*In vivo* sodium tungstate effects. Mice were treated with 2 g/l of sodium tungstate in water for 14 days. Body weight gain (A) and food intake (B) were measured for the duration of treatment, and blood glucose (C) and TG (D) were measured at the beginning and at the end of treatment. Open squares correspond to untreated lean mice (UL) (n = 6), black squares to treated lean mice (TL) (n = 6), open circles to untreated obese mice (UO) (n = 6), and black circles to treated obese mice (TO) (n = 6). In the bar graph, white and black bars represent untreated and treated animals, respectively. *p<0.05; **p<0.01; ***p<0.001 vs. lean mice. ^+++^ *p*<0.001 treated lean vs. untreated lean mice. ^#^ *p*<0.05; ###p<0.001 treated obese vs. untreated obese mice. Data are the mean±SD.

C57Bl6 mice were divided into 4 groups: untreated lean mice (UL); lean mice treated with sodium tungstate (TL); untreated obese mice (UO); and obese mice treated with sodium tungstate (TO). Before sodium tungstate treatment, the obese groups had a significantly higher body weight (data not shown) as compared with the lean animals, and, from the second day of treatment onward, the treated groups showed a significant reduction in body weight gain, maintaining this difference for the entire duration of treatment (Fig. 1A). From the fourth day onward, a significant reduction in food intake in the treated groups was also recorded (Fig. 1B) and maintained for the entire duration of treatment, with no variation in water intake (data not shown). Blood glucose and triglyceride levels were measured before and after treatment. A significant reduction in the values was observed in the obese treated animals (Fig. 1C). The effects of sodium tungstate observed here have been widely described in different models of obesity [14], [15], [19], [27].

### Oral Administration of Sodium Tungstate Modifies the Protein Profile of the Different Hypothalamic Nuclei

We have previously demonstrated that sodium tungstate regulated neuropeptide gene expression in the hypothalamus and neural cells [14], [28]. Therefore, protein expression levels would also be modified using this treatment. To assess whether sodium tungstate could change protein profile expression in the hypothalamus, we performed proteomic analyses of each hypothalamic nucleus individually (ARC, PVN, and LHA).

**Figure 2:**
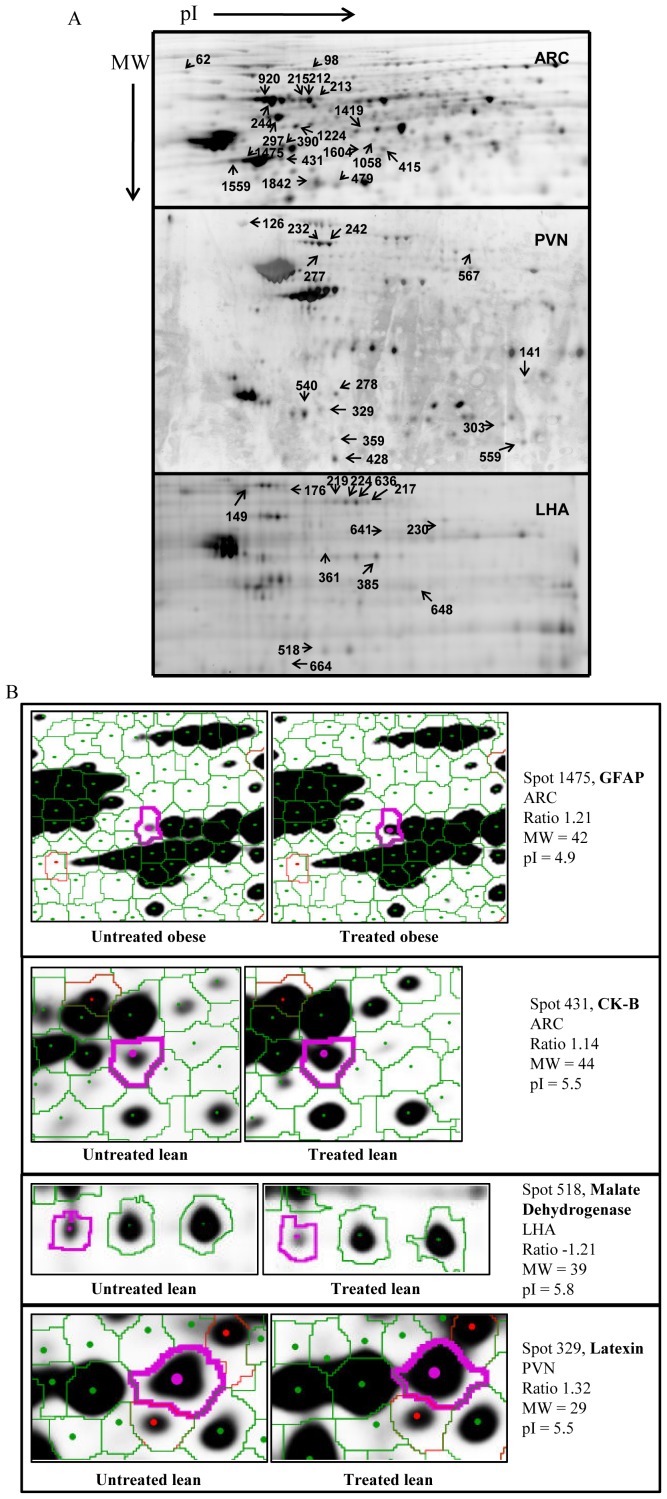
Representative 2D gels from hypothalamic nuclei. Hypothalamic nuclei were isolated and subjected to 2D gel electrophoresis, with the first dimension being the pI from 3 to 10, and the second dimension being the MW resolved in a 10% acrylamide gel. Spot profile analysis was performed using DeCyder software. (A) Representative 2D gels from each hypothalamic nucleus. pI increased from left to right, and MW decreased from top to bottom. All spots exhibiting significant differences (numbered) were selected for protein digestion mass spectrometry, and protein identification summarized in table S1. As an example, a close-up image of differentially expressed proteins between untreated and treated obese mice is shown in (B).

2D-DIGE gels from the UL, TL, UO, and TO groups of animals were done for each nucleus, and spots were analyzed with DeCyder software. All proteins showing significant differences (p<0.05) between compared groups (UL *vs* UO; UL *vs* TL; and UO *vs* TO) were selected (Fig. 2A and close-up images Figure 2B) and picked up to be identified by mass spectrometry. Table 1 shows all identified proteins modified by sodium tungstate. A complete list of protein identification is provided in table S1.

**Table 1:** Tungstate targets identified by proteomics in different hypothalamic nuclei.

Spot^a^	Uniprot	Protein	Phenotype^b^	Ratio^c^	P	Nuclei
**Axonal growth and neuronal plasticity**						
213	O08553	CRMP2	L	1.61	0.011	ARC
215	O08553	CRMP2	L	1.34	0.012	ARC
244	O08553	CRMP2	L	−1.19	0.031	ARC
297	P46660	Alpha-internexin	L	−1.8	0.024	ARC
62	P08553	NEFM	O	1.5	0.037	ARC
1475	P03995	GFAP	O	1.21	0.017	ARC
242	O08553	CRMP2	L	1.34	0.021	PVN
278	P46660	Alpha-internexin	O	−1.1	0.022	PVN
217	O08553	CRMP2	L	−1.32	0.061	LHA
219	O08553	CRMP2	L	−1.41	0.029	LHA
224	O08553	CRMP2	L	−1.61	0.006	LHA
176	O08553	CRMP2	O	1.19	0.059	LHA
**Cytoskeleton and neuronal structure**						
1475	P60710	Beta-actin	O	1.21	0.017	ARC
1559	P60710	Beta-actin	O	1.63	0.028	ARC
1058	Q8BXF8	Actin-relatedprotein M1	O	−1.21	0.047	ARC
1224	P68369	Alpha-tubulin,chain 1A	O	1.61	0.013	ARC
636	A2AQ07	Tubulin beta1	L	−1.81	0.038	LHA
**Energy metabolism**						
415	P14152	Malate dehydrogenase	L	−1.21	0.044	ARC
479	P14152	Malate dehydrogenase	L	1.2	0.027	ARC
1419	P17182	Enolase 1	L	1.36	0.04	ARC
1224	Q8BKZ9	Pyruvate dehydrogenase	O	1.61	0.013	ARC
277	Q9DCT2	NADH dehydrogenase	L	1.72	0.015	PVN
329	Q9DCT2	NADH dehydrogenase	L	1.32	0.02	PVN
303	P17751	Triosephosphate isomerase	O	−1.82	0.023	PVN
567	Q811J3	Aconitase 2	O	−1.77	0.034	PVN
359	Q00623	Apolipoprotein A-I	O	−1.6	0.002	PVN
431	Q04447	Creatine kinase B	L	1.14	0.003	ARC
361	P21550	Enolase 3	L	−1.21	0.028	LHA
373	P17182	Enolase 1	L	−1.62	0.05	LHA
518	P14152	Malate dehydrogenase	L	−1.21	0.023	LHA
664	Q9D051	Pyruvate dehydrogenase-β	L	−1,42	0,049	LHA
230	P52480	Pyruvate kinase	O	1.43	0.015	LHA
1475	Q04447	Creatine kinase B	O	1.21	0.017	ARC
648	P35486	Pyruvate dehydrogenase-α	O	1.61	0.059	LHA
**Others**						
98	P48722	Hspa 4L	L	1.19	0.021	ARC
1604	Q9Z2Q6	Septin-5	L	1.33	0.036	ARC
1224	P62814	Vacuolar H+ATPase B2	O	1.61	0.013	ARC
1058	P31938	MEK1	O	−1.21	0.047	ARC
141	P50518	V- ATPase E1	L	−1.17	0.046	PVN
242	P50516	V-ATPase A	L	1.34	0.021	PVN
329	P70202	Latexin	L	1.32	0.02	PVN
428	Q9R0Y5	Adenilate kinase 1	O	−1.77	0.009	PVN
559	P09671	Superoxide dismutase	O	−1.21	0.047	PVN
149	P63017	Hspa8	L	−1.51	0.052	LHA
664	P62880	Gnb 2	L	−1.42	0.049	LHA

a Spot numbers corresponding to 2D images for each type of nuclei (Figure 2).

b Mice phenotypes: L  =  lean. O  =  obese.

c Ratios of protein expression levels calculated using DeCyder software as the fold change in normalized spot volume comparing tungstate treated and non treated animals (Student’s *t* test based on the log of the ratio of the treated group to the control group).

Analysis of this list highlighted that an important part of the proteins modified by treatment could be classified into three general groups of functions, independently of the nucleus analyzed or the phenotype (lean or obese). These groups of proteins were involved in axonal growth and neuronal plasticity (i.e. alpha-internexin), cytoskeleton and neuronal structure (i.e. β-tubulin) and energy metabolism (i.e. pyruvate dehydrogenase).

### Sodium Tungstate Modifies the Expression of Proteins Involved in Neuronal Plasticity and Axonal Growth

**Figure 3:**
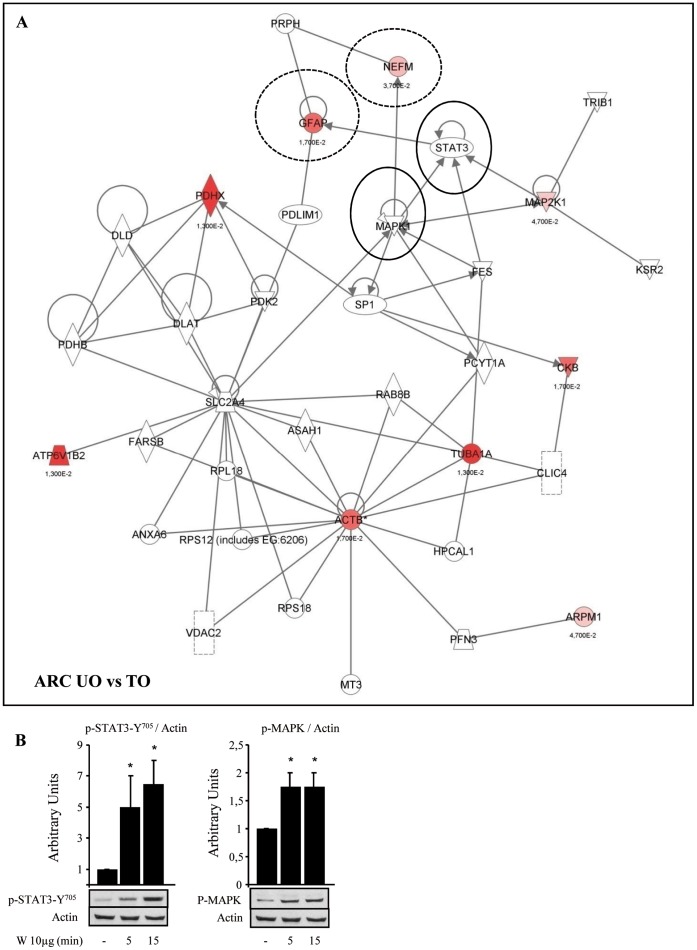
Sodium tungstate effects on the neurofilament protein family. (A) *Ingenuity Pathways* analysis: representative analysis of ARC identified proteins in obese mice (for protein details, see table S1). The network consists of 35 nodes and includes 9 differentially expressed proteins identified by our MS/MS spectra (in red). Note the interactions of GFAP and NEFM (discontinuous circles) with MAPK and STAT3 (black circles). All the identified proteins appeared in the network map. (B) Representative western blot of hypothalamic proteins taken from rats fasted for 8 h, and treated with 10 µg of sodium tungstate (W) via icv (n = 6), and western blot quantification. Tissues were collected 5 and 15 min after treatment. *p<0.05 vs. untreated rats. The leptin signaling pathway was activated in the hypothalamus of sodium tungstate-treated animals as soon as 5 minutes after icv injection, showing changes in the phosphorylation state of STAT3 and MAPK, in accordance with *Ingenuity Pathways* results.

Some proteins belonging to the neurofilament family were identified in the hypothalamus of the treated animals (Table 1): alpha-internexin (INA), neurofilament medium polypeptide (NEFM), and GFAP. All of these proteins are important components of myelinated axons. Using *Ingenuity Pathways Knowledge Base* to identify local networks between experimental proteins and interactions with other proteins in the knowledge base, we defined a strong relationship between NEMF/GFAP and mitogen-activated protein kinase/signal transducer and activator of transcription 3 (MAPK/STAT3) (Fig3A). We further established that sodium tungstate, when centrally administered in the third ventricle, increased the phosphorylation of MAPK and STAT3 in the hypothalamus (Fig. 3B and [28]), confirming the connection between these two proteins and sodium tungstate effects.

**Figure 4:**
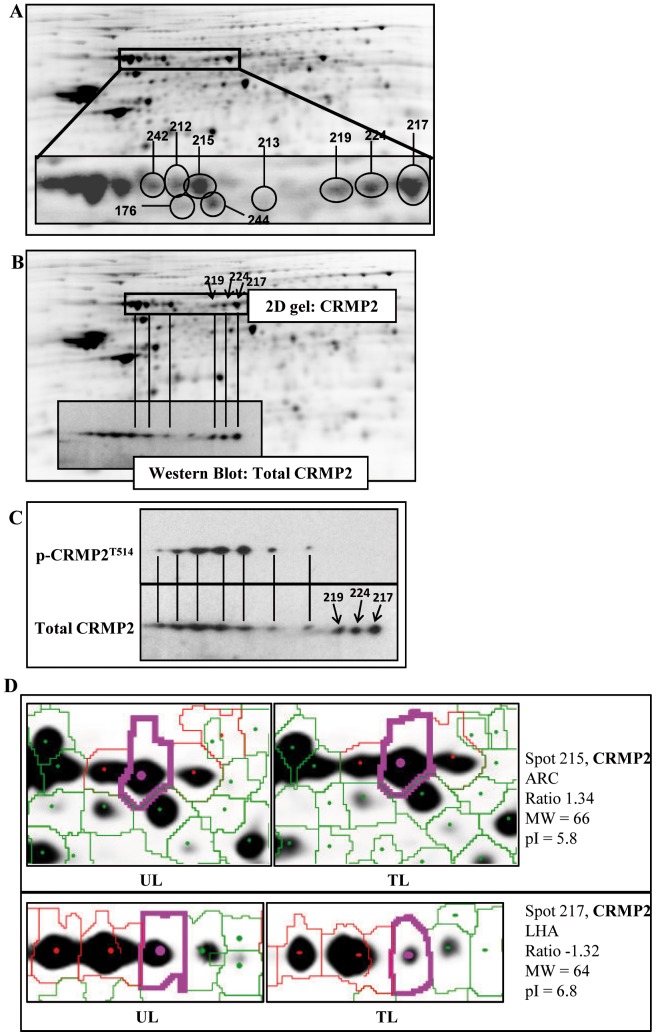
Sodium tungstate has an effect on the CRMP2 protein. An important portion of all spots isolated were identified as CRMP2. (A) Representative ARC 2D gel from treated lean mouse. Insert image shows different CRMP2 proteins. Numbers correspond to identification of CRMP2 by mass spectrometry. (B) Protein identification using mass spectrometry was verified performing 2D-Western blot (insert image). Spots identified as CRMP2 by mass spectrometry co-localized perfectly with those identified by 2D-western blot, confirming that identification had been performed correctly. (C) p-CRMP2^T514^ antibody reveals that a significant number of posttranslational changes of this protein involve phosphorylation, and the great pI variability indicates that multiple posttranslational changes occur all at once. (D) Representative close-up images of spots with different pI corresponding to CRMP2.

Supporting the theory that sodium tungstate could be involved in neuronal structure, another protein implicated in these processes was identified in our analyses, namely CRMP2. CRMP2 was detected in all hypothalamic nuclei analyzed (ARC, PVN, and LHA), and under all different treatment conditions (Table 1, Fig. 4A). This protein binds to tubulin heterodimers to promote microtubule assemblage and cytoskeleton remodeling [29], and it is regulated by phosphoinositide-3 kinase (PI3K), MAPK, and GSK3β [30], [31], [32], proteins which are also modulated by sodium tungstate [33], [34], [35].

Further analysis of the 2D gels showed that all CRMP2 proteins detected corresponded to different spots (Fig. 4A), suggesting multiple posttranslational modifications. To confirm that proteins identified by mass spectrometry actually corresponded to CRMP2, 2D Western blot was performed and total CRMP2 detected (Fig. 4B). The results confirmed the identity of the proteins as CRMP2 (Fig. 4B) and also enabled us to see that there were many more spots corresponding to this protein than originally detected in the 2D gels. The membrane was incubated with pCRMP2-T^514^ antibody (Fig. 4C), and results showed that phosphorylation took place in many spots. This great pI variability could demonstrate that the spots detected in 2D-DIGE gel (Fig. 4D) represent hypo- or hyper-phosphorylated forms of CRMP2, corresponding to basic and acidic pI, respectively and sodium tungstate treatment modulates them (Fig. 4D, spot 217 basic pI decreased and spot 215 acidic pI increased).

Finally, proteomic analysis also showed changes in β-actin, α-tubulin, and β-tubulin (Table 1), all of which are related to the cytoskeleton and to neuronal structure and polarity [36].

### Involvement of Sodium Tungstate in Plasticity-related Reorganization in the ARC Nucleus

**Figure 5:**
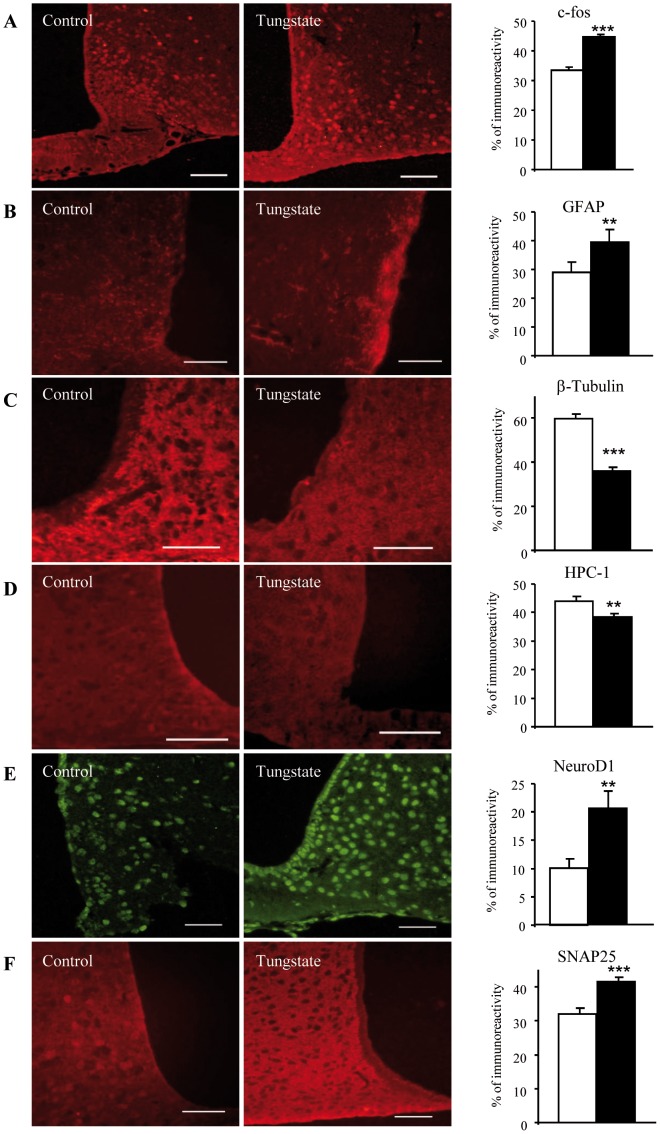
Sodium tungstate treatment significantly modulates expression of proteins involved in neuronal plasticity. Brains from mice fed with a standard diet were sliced following 14 days of tungstate treatment and were prepared for immunoreactivity assays. (A) c-fos antibody reveals an increase in c-fos immunoreactive neurons after tungstate treatment, (B) as does the GFAP antibody. (C) The β-tubulin antibody and (D) HPC-1 show a decrease in immunoreactive neurons for these proteins. (E) NeuroD-1 immunoreactivity and (F) SNAP25 immunoreactivity increase after treatment. Scale bar  = 50 µM. White and black bars represent untreated and treated animals, respectively. **p<0.01; ***p<0.001. Data are the mean±SD.

Further evidence of the effects of sodium tungstate on synaptic plasticity in the hypothalamus was provided by immunohistochemistry studies. There were very few c-fos immunoreactive cells in the ARC of non-treated animals, with no differential expression in the UL and UO animals, as in previous findings [37]. In contrast, the overall number of c-fos immunoreactive cells in the ARC of the treated animals was significantly higher (Fig. 5A). Sodium tungstate also induced morphological modifications in ARC astroglia with changes in the expression and distribution of GFAP, a marker of intermediate filaments in glial cells. The number and size of astroglial profiles in the ARC neuropil exhibited a significant increase (Fig. 5B), which could be related to changes in neuronal coverage through astrocytes and changes in the communication between them. Furthermore, from the list of potential factors identified in our proteomic study, we selected three markers related to axonal growth and neuronal plasticity. In particular, we chose a microtubule protein that has an important role in synaptic plasticity (β-tubulin), CRMP2, and HPC-1 (this last one extracted from *Ingenuity Pathways* analyses, data not shown). In addition, synaptosomal-associated protein 25 (SNAP25), a synaptic protein involved in the regulation of neurotransmitter release, and NeuroD-1, a neurogenic transcription factor involved in plasticity-related reorganization, were also selected for immunohistochemistry studies. Upon quantification of all data, our results showed a reduced expression of β-tubulin (Fig. 5C) and HPC-1 (Fig. 5D) in the ARC of the treated animals, together with an increased expression of NeuroD1 (Fig. 5e) and SNAP25 (Fig. 5F). For total-CRMP2, no clear differences in expression were observed in the treated versus non-treated animals. However, we discovered that the intensity of the immunoreactivity was lower around some ARC neurons in the animals treated with sodium tungstate (data not shown).

All these results suggest that sodium tungstate induces structural and functional remodeling in hypothalamic circuits and that sodium tungstate could be involved in the refinement of ARC circuitry.

### Sodium Tungstate Modifies the PVN Microstructure

**Figure 6:**
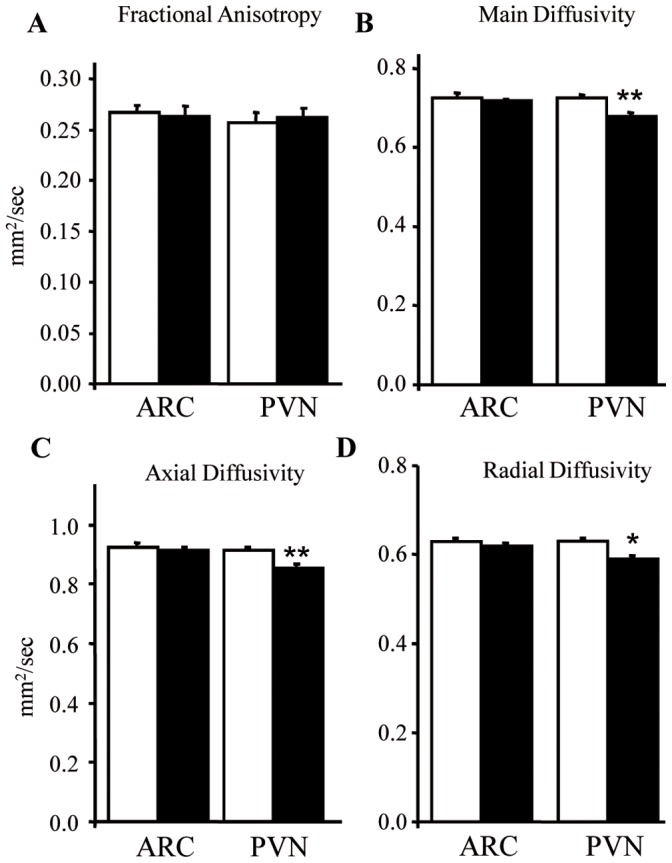
Sodium tungstate modifies neuronal microstructure. Brains from treated and untreated lean mice were analyzed by MRI. (A) Sodium tungstate treatment did not alter fractional anisotropy (FA), neither in the PVN nor in the ARC. In the PVN, treatment decreased: (B) Main diffusivity (MD); (C) axial diffusivity (AD); and (D) radial diffusivity (RD). White and black bars represent untreated and treated animals, respectively. *p<0.05; **p<0.01. Data are the mean±SD.

Finally, to further characterize the effect of sodium tungstate treatment on the hypothalamus, we performed magnetic resonance assays *in vivo*, comparing the ARC and PVN between lean animals treated with sodium tungstate and lean non-treated animals. MRI measures the random diffusional motion of water molecules and provides quantitative indices of the structural features of tissue in central nervous system. DTI is the most promising MRI technique to image changes in white and grey matter integrity, due to their ability to non-invasively assess tissue water diffusion. Different parameters related to brain microstructure can be obtained from DTI images, namely fractional anisotropy (FA), main diffusivity (MD), axial diffusivity (AD) and radial diffusivity (RD) [38], [39], [40]. There were no significant differences in FA values, neither in the ARC nor in the PVN (Fig. 6a). On the contrary, the treatment produced a significant decrease in MD (Fig. 6b), AD (Fig. 6c), and RD (Fig. 6d) in the PVN. However, no significant effects of treatment on MD, AD or RD in the ARC were observed.

These results show a decrease in the movement of water molecules in the PVN, probably due to a higher degree of neuronal organization in the PVN after treatment.

## Discussion

Previous results from our group suggested that sodium tungstate could have direct effects on the central nervous system in rodents, which could partly explain its antiobesity effects [14], [28]. In this report, we thoroughly analyze the effects of sodium tungstate on the hypothalamus, specifically on protein expression, using for this purpose a technique capable to analyze proteins in depth: proteomic analysis.

Results comparing lean and obese animals (non-treated) showed that there were few differences between these two groups, indicating no major variations on protein profile in the hypothalamic nuclei. This fact could be explained by the rapid changes described at the protein level during the initial phase of a high-fat diet (HFD) [41]. Thus, hypothalamic neurons quickly become insulin-resistant, even before animals exhibit a diabetic or obese phenotype [42]. Later on, an adaptive process is triggered and could explain these few differences obtained in our model.

Results comparing treated and non-treated animals showed that sodium tungstate had similar effects on each of the hypothalamic nuclei analyzed (ARC, PVN, and LHA) and on each mouse phenotype (lean or obese), acting principally on proteins involved in energy metabolism, axonal growth, and neuronal plasticity. As described in earlier studies [21], [43], [44], sodium tungstate treatment modulates energy homeostasis in peripheral tissues, with the observed changes not surprisingly also in hypothalamic nuclei. Therefore, we proceeded to focus on the potential effects of the drug on neuronal plasticity, which are less commonly known.

The neuron is one of the most highly polarized cells known, having two structurally and functionally distinct parts, the axon and the dendrites [45]. Elongation of immature neurites is necessary for axon specification. Intracellular mechanisms that help to enhance neurite/axon outgrowth evidently require reorganization of the cytoskeleton, including acting filaments and microtubules [36]. Proteomic and immunohystochemistry results showed that sodium tungstate has significant effects on the neurofilament protein family, essential for the maintenance of neuronal structure [46], [47], [48], and proteins related to cytoskeleton, neuronal structure, and polarity [36], such as β-actin, α-tubulin, and β-tubulin. The function of the neurofilament family of proteins is regulated by MAPK and STAT3. We have demonstrated that sodium tungstate regulates both factors in the hypothalamus, as shown by our icv studies, and in other tissues [28], [34]. GFAP, a characterized marker of intermediate filaments in glial cells, is also regulated by sodium tungstate, as seen in proteomic and immunohistochemistry studies. These findings suggest that the observed changes involve not only ARC neurons but also other neuronal components, such as glial cells. This highlights the importance of the communication between different cell types in order to sustain plastic changes under certain physiological and pathological conditions. In fact, attributing the plasticity of glial cells to their heterogeneity and their delicate sensitivity to neuronal activity and synaptic transmission [49], [50], it is then possible to hypothesize that glial cells could play central roles in the effectiveness of sodium tungstate in reducing body weight gain.

CRMP2 is another key protein for neuronal structure. It is involved in axonal growth and the guidance of immature neurons [51], [52]. Many evidences also support its implication in the pathogenesis of Alzheimer’s disease [53], [54], [55]. Sodium tungstate plays an important role in CRMP2 regulation, as it induced changes at the post-translational level in all hypothalamic nuclei and mouse phenotypes analyzed, although no changes in total protein were observed in immunohistochemistry studies. CRMP2 is regulated by Pi3K, MAPK, and GSK3-β [30], [31], [32], all of which are regulated, in turn, by sodium tungstate [33], [34], [35]. Therefore, even though further studies on the specific effects of sodium tungstate on CRMP2 activity and post-translational modifications are needed, here we establish a strong relationship between them.

The immunohistochemistry results with β-tubulin, HPC-1, NeuroD1 and c-fos show that sodium tungstate induced structural and functional remodeling in the hypothalamic circuits, regulating food intake, and neuronal activation. Since c-fos immunoreactivity is not usually elicited by steady state conditions, the elevated levels of c-fos in the ARC nucleus of the treated animals may reflect some kind of adaptation involving central food intake regulation, nutrient absorption, thermogenesis, and fat metabolism or storage [56], [57].

Proteomics and immunohistochemistry results would seem to indicate some discrepancies for some proteins, i.e. HPC-1, NeuroD1, SNAP25 or b-tubulin. 2D-DIGE is currently one of the most powerful quantitative high-throughput proteomics technologies available [58], which allows for the quantisation of hundreds and even thousands of proteins under different conditions at once. Like all non-targeted proteomics technologies, one of the limitations of all these approaches, both gel-based and gel-free (LC-MSMS), is that less abundant proteins might be masked by more abundant ones. In addition, when using 2D, one protein can be split in several spots (with different mw/pI as observed in our study), which sometimes make an overall protein quantification difficult. Thus, the fact that one particular protein may not be observed in the analyses does not imply that this protein may be changed under the conditions studied. This could be the case of b-tubulin in ARC. Another limitation of 2D electrophoresis is the difficulty in dealing with high and low molecular weight proteins, extreme pI and hydrophobic membrane proteins. The latter is indeed the case of SNAP25 and HPC-1, both components of the SNARE complex and membrane and synaptosome components. Thus, it is likely that these proteins may not enter in the IPG strip of the 1^st^ dimension of 2D gel. Nevertheless, HPC-1 was detected by the IPA analysis, which is a curated database and it is used for validation of proteomics results. NeuroD1 is a transcription factor and, as with most transcription factors, it is probably expressed at low concentrations in a whole tissue homogenate. Therefore, its detection by DIGE and other non-targeted proteomic approaches is also difficult.

MRI analysis gave us further evidence that sodium tungstate might actually have a role in neuronal plasticity: changes in water diffusivity are related with changes in microstructure. FA is sensitive to water motion along myelinated axons, rather than perpendicular to them. Since mice brains have little white matter as compared to human brains, this index is less accurate in rodent studies. MD is a global measure of water motion, and it increases after neuronal damage, since edema and loss of neurons involve higher water content in the tissue. AD reflects diffusion of water parallel to the axon, while RD reflects diffusion perpendicular to the axon. Thus, an increase in AD and RD reflects axonal damage and demyelination, respectively. On the other hand, an increase in AD and RD has been associated to axonal regeneration and remyelination [59], [60]. Although we could not validate our DTI findings with histology, based on the other evidences described in this study, we suggest that the decrease observed in AD and RD after sodium tungstate treatment in the PVN might reflect reorganization of the structure possibly related to axonal sprouting.

The effects of sodium tungstate on neuronal plasticity may have important implications and potential applications. Animals predisposed to develop obesity on a high-fat diet (which are leptin resistant) have defective neuronal connections [6], [8], [61], [62]. Leptin has also been described as playing an important role in neuronal development and in the projections between the different hypothalamic nuclei during the early postnatal period, and it has been described that sodium tungstate acts on leptin pathway [16]. Our proteomic, immunohistochemistry and MRI results give important clues about possible courses of action of sodium tungstate on the regulation of energy metabolism, and all these are promising future outcomes of this treatment. We also open the door to studying sodium tungstate actions on other neurological diseases like Alzheimer’s disease.

The fact that sodium tungstate could modulate neural plasticity in adult animals makes it a suitable and promising candidate for improving obesity management in adult populations.

## Supporting Information

Table S1 **Tungstate targets identified by proteomics in different hypothalamic nuclei.**
(DOC)

## References

[1] Morton GJ, Cummings DE, Baskin DG, Barsh GS, Schwartz MW (2006). Central nervous system control of food intake and body weight.. Nature.

[2] Vianna CR, Coppari R (2011). A Treasure Trove of Hypothalamic Neurocircuitries Governing Body Weight Homeostasis.. Endocrinology.

[3] Zheng H, Berthoud H-R (2008). Neural Systems Controlling the Drive to Eat: Mind Versus Metabolism.. Physiology.

[4] Dietrich MO, Horvath TL (2009). Feeding signals and brain circuitry.. European Journal of Neuroscience.

[5] Cone RD (2005). Anatomy and regulation of the central melanocortin system.. Nat Neurosci.

[6] Bouret SG, Gorski JN, Patterson CM, Chen S, Levin BE (2008). Hypothalamic Neural Projections Are Permanently Disrupted in Diet-Induced Obese Rats.. Cell Metabolism.

[7] Pinto S, Roseberry AG, Liu H, Diano S, Shanabrough M (2004). Rapid rewiring of arcuate nucleus feeding circuits by leptin.. Science.

[8] Bouret S, Draper SJ, Simerly RB (2004). Trophic action of leptin on hypothalamic neurons that regulate feeding.. Science.

[9] Andrews ZB, Liu ZW, Walllingford N, Erion DM, Borok E (2008). UCP2 mediates ghrelin’s action on NPY/AgRP neurons by lowering free radicals.. Nature.

[10] Fontán-Lozano Á, López-Lluch G, Delgado-García J, Navas P, Carrión Á (2008). Molecular Bases of Caloric Restriction Regulation of Neuronal Synaptic Plasticity.. Molecular Neurobiology.

[11] Horvath TL, Diano S, Tschöp M (2004). Brain Circuits Regulating Energy Homeostasis.. The Neuroscientist.

[12] Stankiewiez PaG, MJ (1988). Inhibition of phosphatases and sulfatases by transition-state analogues.. Biochemistry.

[13] Kletzin AMA (1996). Tungsten in biological systems.. FEMS Microbiol Rev.

[14] Canals I, Carmona MC, Amigo-Correig M, Barbera A, Bortolozzi A (2008). A functional leptin system is essential for sodium tungstate antiobesity action.. Endocrinology.

[15] Claret M, Corominola H, Canals I, Saura J, Barcelo-Batllori S (2005). Tungstate Decreases Weight Gain and Adiposity in Obese Rats through Increased Thermogenesis and Lipid Oxidation.. Endocrinology.

[16] Amigó-Correig M, Barceló-Batllori S, Piquer S, Soty M, Pujadas G (2011). Sodium tungstate regulates food intake and body weight through activation of the hypothalamic leptin pathway.. Diabetes, Obesity and Metabolism.

[17] Gómez-Ramos A, Avila JDDZHCRGJJGJ (2006). Sodium tungstate decreases the phosphorylation of tau through GSK3 inactivation.. Journal of Neuroscience Research.

[18] Enriori PJ, Evans AE, Cowley MA (2007). Diet-induced obesity causes severe but reversible leptin resistance in arcuate melanocortin neurons.. Cell Metabolism.

[19] Barcelo-Batllori S, Kalko SG, Esteban Y, Moreno S, Carmona MC (2008). Integration of DIGE and Bioinformatics Analyses Reveals a Role of the Antiobesity Agent Tungstate in Redox and Energy Homeostasis Pathways in Brown Adipose Tissue.. Mol Cell Proteomics.

[20] Shevchenko A, Wilm M, Vorm O, M M (1996). Mass spectrometric sequencing of proteins silver-stained polyacrylamide gels.. Anal Chem.

[21] Barcelo-Batllori S, Corominola H, Claret M, Canals I, Guinovart J (2005). Target identification of the novel antiobesity agent tungstate in adipose tissue from obese rats.. PROTEOMICS.

[22] Barceló-Batllori S, André M, Servis C, Lévy N, Takikawa O (2002). Proteomic analysis of cytokine induced proteins in human intestinal epithelial cells: Implications for inflammatory bowel diseases.. PROTEOMICS.

[23] Felley-Bosco E, Demalte I, Barcelo S, Sanchez J-C, Hochstrasser DF (1999). Information transfer between large and small two-dimensional polyacrylamide gel electrophoresis.. Electrophoresis.

[24] Taylor CR, Laevenson RM (2006). Quantification of immunohistochemistry-issues concerning methods, utility and semiquantitative assessment II.. Histopathology.

[25] Lazar M, Lee J, Alexander A (2005). Axial asymmetry of water diffusion in brain white matter.. Magnetic Resonance in Medicine.

[26] Hasan K, Narayana P (2006). Retrospective measurement of the diffusion tensor eigenvalues from diffusion anisotropy and mean difusivity in DTI.. Magnetic Resonance in Medicine.

[27] Munoz MC, Barbera A, Dominguez J, Fernandez-Alvarez J, Gomis R (2001). Effects of Tungstate, a New Potential Oral Antidiabetic Agent, in Zucker Diabetic Fatty Rats.. Diabetes.

[28] Amigó-Correig M, Barceló-Batllori S, Piquer S, Soty M, Pujadas G, et al. Sodium tungstate regulates food intake and body weight through activation of the hypothalamic leptin pathway.. Diabetes, Obesity and Metabolism In press.

[29] Fukata Y, Itoh TJ, Kimura T, Menager C, Nishimura T (2002). CRMP-2 binds to tubulin heterodimers to promote microtubule assembly.. Nat Cell Biol.

[30] Yoshimura T, Arimura N, Kawano Y, Kawabata S, Wang S (2006). Ras regulates neuronal polarity via the PI3-kinase/Akt/GSK-3[beta]/CRMP-2 pathway.. Biochemical and Biophysical Research Communications.

[31] Yoshimura T, Kawano Y, Arimura N, Kawabata S, Kikuchi A (2005). GSK-3[beta] Regulates Phosphorylation of CRMP-2 and Neuronal Polarity.. Cell.

[32] Arimura N, Menager C, Kawano Y, Yoshimura T, Kawabata S (2005). Phosphorylation by Rho kinase regulates CRMP-2 activity in growth cones.. Mol Cell Biol 25.

[33] Alberto G-R, Jorge D, Delia Z, Helena C, Ramon G (2006). Sodium tungstate decreases the phosphorylation of tau through GSK3 inactivation.. Journal of Neuroscience Research.

[34] Carmona MC, Amigo M, Barcelo-Batllori S, Julia M, Esteban Y (2009). Dual effects of sodium tungstate on adipocyte biology: inhibition of adipogenesis and stimulation of cellular oxygen consumption.. Int J Obes.

[35] Piquer S, Barcelo-Batllori S, Julia M (2007). Phosphorylation events implicating p38 and PI3K mediate tungstate-effects in MIN6 beta cells Biochem Biophys Res Commun.

[36] Brown A, Slaughter T, Black MM (1992). Newly assembled microtubules are concentrated in the proximal and distal regions of growing axons.. The Journal of Cell Biology.

[37] Becskei C, Lutz TA, Riediger T (2009). Blunted Fasting-Induced Hypothalamic Activation and Refeeding Hyperphagia in Late-Onset Obesity.. Neuroendocrinology.

[38] Pierpaoli C, Basser PJ (1996). Toward a quantitative assessment of diffusion anisotropy.. Magnetic Resonance in Medicine.

[39] Basser PJ, Pierpaoli C (1996). Microstructural and Physiological Features of Tissues Elucidated by Quantitative-Diffusion-Tensor MRI.. Journal of Magnetic Resonance, Series B.

[40] Beaulieu C (2002). The basis of anisotropic water diffusion in the nervous system – a technical review.. NMR in Biomedicine.

[41] Banas S, Rouch C, Kassis N (2009). A dietary fat excess alters metabolic and neuroendocrine responses before the onset of metabolic diseases.. Cell Mol Neurobiol.

[42] Ono H, Pocai A, Wang Y, Sakoda H, Asano T (2008). Activation of hypothalamic S6 kinase mediates diet-induced hepatic insulin resistance in rats.. The Journal of Clinical Investigation.

[43] Barbera A, Rodriguez-Gil JE, Guinovart JJ (1994). Insulin-like actions of tungstate in diabetic rats. Normalization of hepatic glucose metabolism.. J Biol Chem.

[44] Barbera AGR, Prats N, Rodriguez-Gil JE, Domingo M, Gomis R (2001). Tungstate is an effective antidiabetic agent in streptozotocin-induced rat: a long term study.. Diabetologia 44.

[45] Craig AM, Banker G (1994). Neuronal polarity.. Annual Review of Neuroscience.

[46] Veeranna G, Shetty K (2000). Cdk5 and MAPK are associated with complexes of cytoskeletal proteins in rat brain.. Molecular Brain Research.

[47] Evans J, Sumners C, Moore J, Huentelman MJ, Deng J (2002). Characterization of Mitotic Neurons Derived From Adult Rat Hypothalamus and Brain Stem.. J Neurophysiol.

[48] Sihag R, Inagaki M (2007). Role of phosphorylation on the structural dynamics and function of types III and IV intermediate filaments Experimental Cell Research.

[49] Todd KJ, Serrano A, Lacaille J-C, Robitaille R (2006). Glial cells in synaptic plasticity.. Journal of Physiology-Paris.

[50] Bernabeu A, Alfaro A, García M, Fernández E (2009). Proton magnetic resonance spectroscopy (1H-MRS) reveals the presence of elevated myo-inositol in the occipital cortex of blind subjects.. NeuroImage.

[51] Inagaki N, Chihara K, Arimura N, Menager C, Kawano Y (2001). CRMP-2 induces axons in cultured hippocampal neurons.. Nat Neurosci.

[52] Yamashita N, Ohshima T, Nakamura F, Kolattukudy P, Honnorat J (2012). Phosphorylation of CRMP2 (Collapsin Response Mediator Protein 2) Is Involved in Proper Dendritic Field Organization.. The journal of neuroscience.

[53] Charrier E, Reibel S, Rogemond V, Aguera M, Thomasset N (2003). Collapsin response mediator proteins (CRMPs).. Molecular Neurobiology.

[54] Gu Y, Hamajima N, Ihara Y (2000). Neurofibrillary Tangle-Associated Collapsin Response Mediator Protein-2 (CRMP-2) Is Highly Phosphorylated on Thr-509, Ser-518, and Ser-522.. Biochemistry.

[55] Kanninen K, Goldsteins G, Auriola S, Alafuzoff I, Koistinaho J (2004). Glycosylation changes in Alzheimer’s disease as revealed by a proteomic approach.. Neuroscience Letters.

[56] Verty ANA, Allen AM, Oldfield BJ (2010). The Endogenous Actions of Hypothalamic Peptides on Brown Adipose Tissue Thermogenesis in the Rat.. Endocrinology.

[57] Mungarndee S, Lundy RJ, Norgren R (2008). Expression of Fos during sham sucrose intake in rats with central gustatory lesions.. Am J Physiol Regul Integr Comp Physiol.

[58] Marouga R DS, Hawkins E (2005). The development of the DIGE system: 2D fluorescence difference gel analysis technology.. Anal Bioanal Chem.

[59] Tobin JE, Xie M, Le TQ, Song S-K, Armstrong RC (2011). Reduced Axonopathy and Enhanced Remyelination After Chronic Demyelination in Fibroblast Growth Factor 2 (Fgf2)-Null Mice: Differential Detection With Diffusion Tensor Imaging.. Journal of Neuropathology & Experimental Neurology 70: 157–165 110.1097/NEN.1090b1013e31820937e31820934.

[60] Morisaki S, Kawai Y, Umeda M, Nishi M, Oda R (2011). In vivo assessment of peripheral nerve regeneration by diffusion tensor imaging.. Journal of Magnetic Resonance Imaging.

[61] Harris RBS, Kasser TR, Martin RJ (1986). Dynamics of Recovery of Body Composition After Overfeeding, Food Restriction or Starvation of Mature Female Rats.. J Nutr.

[62] Bouret SG, Draper SJ, Simerly RB (2004). Formation of Projection Pathways from the Arcuate Nucleus of the Hypothalamus to Hypothalamic Regions Implicated in the Neural Control of Feeding Behavior in Mice.. J Neurosci.

