# Supplementary material for: Anti-Obesity Sodium Tungstate Treatment Triggers Axonal and Glial Plasticity in Hypothalamic Feeding Centers
Source: PLoS One. 2012 Jul 3;7(7):e39087. doi: 10.1371/journal.pone.0039087 (PMC3389016; doi:10.1371/journal.pone.0039087)
Supplement: Table S1 — Tungstate targets identified by proteomics in different hypothalamic nuclei. (DOC) [file pone.0039087.s001.doc]

**Supplementary table S1**. Tungstate targets identified by proteomics in different hypothalamic nuclei.

|  |  |  | | | **Observed in 2D gel** | | | **Uniprot reference** | |  |  | **Protein** | **Peptide** | **MS/MS peptide** | **% sequence** |
| --- | --- | --- | --- | --- | --- | --- | --- | --- | --- | --- | --- | --- | --- | --- | --- |
| ***Spot^a^*** | **UniProt** | **Protein** | | | **MW** | **pI** | | **MW** | **pI** | **Ratio^b^** | **P-value** | **mascot score^c^** | **number^d^** | **number** | **coverage** |
| **ARC. Untreated lean vs Untreated obese** | | |  | | | |  | | |  |  |  |  |  |  |
| 212 | O08553 | CRMP2 | | | 66 | 5,7 | | 62,5 | 5,95 | 1,3 | 0,026 | 173 | 5 | 4 | 11 |
| 390 | Q9CZ13 | Cytochrome b-c1 | | | 50 | 5,6 | | 53,92 | 5,75 | 1,18 | 0,0077 | 829 | 20 | 18 | 35 |
| 920 | P63017 | Hspa8 | | | 66 | 5,1 | | 70,98 | 5,37 | 1,4 | 0,07 | 1130 | 24 | 23 | 39 |
| 1842 | P16125 | Lactate dehydrogenase | | | 38 | 5,5 | | 36,83 | 5,7 | 1,42 | 0,064 | 452 | 14 | 13 | 26 |
| **ARC. Untreated lean vs Treated lean** | | | |  | | | |  |  |  |  |  |  |  |  |
| 98 | P48722 | APG-1 | | | 80 | 5,8 | | 95,28 | 5,54 | 1,19 | 0,021 | 606 | 20 | 18 | 22 |
| 213 | O08553 | CRMP2 | | | 66 | 5,6 | | 62,63 | 5,95 | 1,61 | 0,011 | 363 | 10 | 7 | 21 |
| 215 | O08553 | CRMP2 | | | 66 | 5,8 | | 62,5 | 5,95 | 1,34 | 0,012 | 83 | 5 | 3 | 11 |
| 244 | O08553 | CRMP2 | | | 66 | 5,3 | | 62,5 | 5,95 | -1,19 | 0,031 | 1250 | 27 | 26 | 40 |
| 297 | P46660 | Alpha-internexin | | | 58 | 5,3 | | 55,52 | 5,23 | -1,8 | 0,024 | 1730 | 27 | 27 | 46 |
| 415 | P14152 | Malate dehydrogenase | | | 44 | 6,5 | | 36,62 | 6,16 | -1,21 | 0,044 | 74 | 4 | 3 | 10 |
| 431 | Q04447 | Creatine kinase B | | | 44 | 5,5 | | 42,93 | 5,4 | 1,14 | 0,0029 | 784 | 18 | 17 | 34 |
| 479 | P14152 | Malate dehydrogenase | | | 38 | 6 | | 36,62 | 6,16 | 1,2 | 0,027 | 350 | 12 | 11 | 26 |
| 1419 | P17182 | Enolase 1 | | | 50 | 6,4 | | 51,7 | 6,37 | 1,36 | 0,04 | 848 | 16 | 15 | 47 |
| 1604 | Q9Z2Q6 | Septin-5 | | | 40 | 6,1 | | 36,6 | 6,21 | 1,33 | 0,036 | 272 | 6 | 5 | 26 |
| **ARC. Untreated obese vs Treated obese** | | | | |  |  | |  |  |  |  |  |  |  |  |
| 62 | P08553 | Nemf | | | 95 | 4 | | 95,99 | 4,76 | 1,5 | 0,037 | 702 | 15 | 15 | 18 |
| 1224 | P62814 | Vacuolar H+ATPase B2 | | | 50 | 5,5 | | 56,85 | 5,57 | 1,61 | 0,013 | 457 | 11 | 10 | 24 |
|  | P68369 | Alpha-tubulin, chain 1A | | |  |  | | 50,81 | 4,94 |  |  | 282 | 9 | 8 | 26 |
|  | Q8BKZ9 | Pyruvate dehydrogenase | | |  |  | | 54,25 | 7,62 |  |  | 155 | 5 | 5 | 11 |
| 1475 | P60710 | Beta-actin | | | 42 | 4,9 | | 42,19 | 5,29 | 1,21 | 0,017 | 562 | 16 | 11 | 38 |
|  | Q04447 | Creatine kinase B | | |  |  | | 42,97 | 5,4 |  |  | 203 | 4 | 4 | 13 |
|  | P03995 | GFAP | | |  |  | | 48,49 | 5,28 |  |  | 201 | 4 | 4 | 10 |
| 1058 | Q8BXF8 | Actin-related protein 1 homologue B | | | 50 | 6,7 | | 42,36 | 5,43 | -1,21 | 0,047 | 519 | 14 | 11 | 47 |
|  | P31938 | MEK1 | | |  |  | | 43,79 | 6,24 |  |  | 170 | 9 | 7 | 22 |
| 1559 | P60710 | Beta-actin | | | 44 | 4,9 | | 42,12 | 5,29 | 1,63 | 0,028 | 188 | 6 | 4 | 31 |
| **PVN. Untreated lean vs Untreated obese** | | | | |  |  | |  |  |  |  |  |  |  |  |
| 232 | [P63018](http://www.uniprot.org/uniprot/P63017) | Hspa8 | | | 66 | 5,5 | | 71 | 5,37 | 1,19 | 0,056 | 160 | 4 | 4 | 5 |
| 360 | Q00624 | Apolipoprotein A-I | | | 28 | 5,3 | | 30,3 | 5,64 | 1,21 | 0,0021 | 426 | 11 | 10 | 34 |
| 428 | Q9R0Y6 | Adenylate kinase isoenzyme 1 | | | 26 | 5,6 | | 23,3 | 5,67 | 1,51 | 0,0049 | 487 | 13 | 13 | 36 |
| **PVN. Untreated lean vs Treated lean** | | | | |  |  | |  |  |  |  |  |  |  |  |
| 141 | [P50518](http://www.uniprot.org/uniprot/P50518) | V- ATPase E1 | | | 30 | 8,2 | | 26,2 | 8,44 | -1,17 | 0,046 | 494 | 18 | 16 | 41 |
| 242 | [O08553](http://www.uniprot.org/uniprot/O08553) | CRMP2 | | | 66 | 5,6 | | 62,6 | 5,95 | 1,34 | 0,021 | 386 | 11 | 8 | 21 |
|  | [P50516](http://www.uniprot.org/uniprot/P50516) | V- ATPase A | | |  |  | | 68,56 | 5,42 |  |  | 288 | 11 | 9 | 19 |
| 277 | [Q9DCT2](http://www.uniprot.org/uniprot/Q9DCT2) | NADH dehydrogenase, mitochondrial | | | 30 | 5,4 | | 30,3 | 6,67 | 1,72 | 0,015 | 346 | 7 | 7 | 30 |
| 329 | [Q00623](http://www.uniprot.org/uniprot/Q00623) | Apolipoprotein A-I | | | 29 | 5,5 | | 31 | 5,64 | 1,32 | 0,02 | 108 | 2 | 2 | 14 |
|  | [Q9DCT2](http://www.uniprot.org/uniprot/Q9DCT2) | NADH dehydrogenase, mitochondrial | | |  |  | | 30 | 6,67 |  |  | 109 | 3 | 3 | 10 |
|  | [P70202](http://www.uniprot.org/uniprot/P70202) | Latexin | | |  |  | | 25,6 | 5,48 |  |  | 83 | 2 | 2 | 9 |
| **PVN. Untreated obese vs Treated obese** | | | | |  |  | |  |  |  |  |  |  |  |  |
| 232 | [P63017](http://www.uniprot.org/uniprot/P63017) | Hspa8 | | | 66 | 5,5 | | 71 | 5,37 | 1,4 | 0,068 | 160 | 4 | 4 | 5 |
| 278 | [P46660](http://www.uniprot.org/uniprot/P46660) | Alpha-internexin | | | 55 | 5,5 | | 55,5 | 5,23 | -1,1 | 0,022 | 1140 | 27 | 24 | 37 |
| 303 | [P17751](http://www.uniprot.org/uniprot/P17751) | Triosephosphate isomerase | | | 28 | 8 | | 27 | 6,9 | -1,82 | 0,023 | 645 | 13 | 13 | 43 |
| 359 | Q00623 | Apolipoproteina A-I | | | 28 | 5,3 | | 30,3 | 5,64 | -1,6 | 0,0021 | 425 | 11 | 10 | 32 |
| 428 | Q9R0Y5 | Adenilate kinase, isoenzime 1 | | | 26 | 5,6 | | 23,3 | 5,67 | -1,77 | 0,0092 | 490 | 13 | 13 | 39 |
| 540 | [P61982](http://www.uniprot.org/uniprot/P61982) | 14-3-3 protein gamma | | | 30 | 4,5 | | 27,9 | 4,8 | 1,16 | 0,06 | 634 | 21 | 21 | 51 |
|  | [P63101](http://www.uniprot.org/uniprot/P63101) | Superoxide dismutase, mitochondrial | | |  |  | | 27,9 | 4,73 |  |  | 504 | 19 | 14 | 47 |
| 567 | [Q811J3](http://www.uniprot.org/uniprot/Q811J3) | Aconitase 2, mitochondrial | | | 80 | 7,8 | | 86,1 | 6,41 | -1,77 | 0,034 | 563 | 14 | 14 | 5 |
| **LHA. Untreated lean vs Untreated obese** | | | | |  |  | |  |  |  |  |  |  |  |  |
| 641 | Q9D0F9 | Phosphoglucomutase-1 | | | 60 | 6,5 | | 61,7 | 6,3 | 1,9 | 0,0023 | 572 | 14 | 12 | 20 |
| **LHA. Untreated lean vs Treated lean** | | | | |  |  | |  |  |  |  |  |  |  |  |
| 149 | P63017 | Hspa8 | | | 70 | 5 | | 71,02 | 5,37 | -1,51 | 0,052 | 916 | 24 | 23 | 33 |
| 217 | [O08553](http://www.uniprot.org/uniprot/O08553) | CRMP2 | | | 64 | 6,8 | | 62,63 | 5,95 | -1,32 | 0,061 | 533 | 9 | 9 | 20 |
| 219 | [O08553](http://www.uniprot.org/uniprot/O08553) | CRMP2 | | | 64 | 6,1 | | 62,63 | 5,95 | -1,41 | 0,029 | 667 | 21 | 18 | 32 |
| 224 | [O08553](http://www.uniprot.org/uniprot/O08553) | CRMP2 | | | 64 | 6,2 | | 62,63 | 5,95 | -1,61 | 0,0064 | 696 | 14 | 14 | 25 |
| 361 | P21550 | Enolase 3 | | | 45 | 6,1 | | 47,3 | 6,73 | -1,21 | 0,028 | 167 | 4 | 4 | 26 |
| 373 | P17182 | Enolase 1 | | | 45 | 7 | | 47,4 | 6,37 | -1,62 | 0,052 | 509 | 9 | 9 | 22 |
| 385 | P17182 | Enolase 1 | | | 45 | 6,8 | | 47,4 | 6,37 | -1,41 | 0,055 | 958 | 32 | 32 | 44 |
| 518 | P14152 | Malate dehydrogenase cytoplasmic | | | 39 | 5,8 | | 36,6 | 6,16 | -1,21 | 0,023 | 293 | 8 | 7 | 24 |
| 636 | A2AQ07 | Beta tubulin | | | 66 | 6,5 | | 57,9 | 4,96 | -1,81 | 0,033 | 79 | 2 | 2 | 6 |
| 664 | P62880 | Gnb2 | | | 39 | 5,4 | | 38 | 5,6 | -1,42 | 0,049 | 497 | 14 | 14 | 26 |
|  | Q9D051 | Pyruvate dehydrogenase E1 beta | | |  |  | | 35,1 | 6,41 |  |  | 294 | 8 | 5 | 23 |
| **LHA. Untreated obese vs Treated obese** | | | | |  |  | |  |  |  |  |  |  |  |  |
| 176 | O08553 | CRMP2 | | | 68 | 5,6 | | 62,6 | 5,95 | 1,33 | 0,049 | 441 | 13 | 8 | 19 |
| 230 | P52480 | Pyruvate kinase | | | 60 | 7 | | 58,3 | 7,17 | 1,43 | 0,015 | 510 | 12 | 11 | 22 |
| 648 | P15105 | Glutamine synthetase | | | 40 | 7,2 | | 42,9 | 6,64 | 1,61 | 0,059 | 273 | 6 | 6 | 13 |
|  | P35486 | Pyruvate dehydrogenase E1, mitochondrial | | |  |  | | 43,8 | 8,49 |  |  | 104 | 2 | 2 | 6 |

***^a^*** Spot numbers corresponding to 2D images for each type of nuclei (figure 2).

***^b^*** Ratios of protein expression levels calculated using DeCyder software as the fold change in normalized spot volume comparing tungstate treated and non treated animals (Student’s *t* test based on the log of the ratio of the treated group to the control group).

***^c^*** Score obtained using the identifying Mascot search engine.

***^d^*** total number of nonredundant peptides assigned to the protein.
